# Herbal therapies in gastrointestinal and hepatic disorders: An evidence-based clinical review

**DOI:** 10.3389/fphar.2022.962095

**Published:** 2022-10-05

**Authors:** Yongfang Yao, Murad Habib, Hajra Fazeelat Bajwa, Anina Qureshi, Rameesha Fareed, Reem Altaf, Umair Ilyas, Yongtao Duan, Muhammad Abbas

**Affiliations:** ^1^ Henan Provincial Key Laboratory of Children’s Genetics and Metabolic Diseases, Children’s Hospital Affiliated to Zhengzhou University, Zhengzhou University, Zhengzhou, Henan, China; ^2^ Ministry of Education of China, Key Laboratory of Advanced Drug Preparation Technologies, Zhengzhou University, Zhengzhou, Henan, China; ^3^ Medical School, Huanghe Science and Technology University, Zhengzhou, Henan, China; ^4^ Department of Paediatric Surgery, The Children’s Hospital, Pakistan Institute of Medical Sciences, Islamabad, Pakistan; ^5^ Islamabad Medical & Dental College, Islamabad, Pakistan; ^6^ Margalla College of Pharmacy, Margalla Institute of Health Sciences, Rawalpindi, Pakistan; ^7^ Riphah Institute of Pharmaceutical Sciences, Riphah International University, Islamabad, Pakistan; ^8^ Department of Pharmacy, Iqra University Islamabad Campus, Islamabad, Pakistan

**Keywords:** ethnopharmacology, herbal medication, GIT, liver, treatment

## Abstract

The gastrointestinal tract (GIT) and the liver constitute the major organs of the human body. Indeed, the very survival of the human body depends on their proper functioning. Because the GIT is a huge and complex organ system, the maintenance of proper GIT and liver health is an arduous task. GIT disturbances such as diarrhea, stomach ache, flatulence, constipation, nausea, and vomiting are very common, and they contribute to a significant burden on the healthcare system. Pharmacies are full of over-the-counter pharmacological drugs to alleviate its common conditions. However, these drugs do not always prove to be fully effective and patients have to keep on living with these ailments without a proper and long-term solution. The aim of this review article is to present a practical reference guide to the role of herbal medicines in dealing with gastrointestinal and hepatic disorders, which is supported by systematic reviews and evidence-based trials. People have depended on herbal medications for centuries for the treatment of various ailments of the GIT, liver, and other organ system problems. Recently, this trend of incorporating herbal medication for the treatment of various diseases in both developing and developed countries have surged. Many people continue to use herbal medications, even though substantial data about their efficacy, uses, and toxicological effects do not exist. In addition, while herbal medicines have enormous benefits in both the prevention and the treatment of medical ailments, they can also have toxicological effects. It is, therefore, of the utmost importance that appropriate time, energy, and resources are spent on the development of ethnopharmacology. In addition, herbal products should be classified in a pattern similar to pharmacological medications, including their uses, side effects, mechanism of action, efficacy, and so on.

## Introduction

The gastrointestinal tract (GIT) is one of the largest and most functionally vital organ systems of the human body. It consists of many components, including the alimentary canal, salivary glands, the pancreas, and the liver (Hu et al., 2022). All these organ systems within the GIT work in collaboration to bring about the process of digestion, absorption, and excretion after the food is ingested through the mouth (Hornbuckle et al., 2008). GIT discomfort is a very common problem and many people prefer to use herbal ingredients to alleviate these disorders (Campanella et al., 2022). Common GIT disorders such as nausea, vomiting, diarrhea, irritable bowel disease, and so on have all been effectively treated with the use of herbal medicines (Campanella et al., 2022). Functional GIT disorders—namely, gastroesophageal reflux disease, dyspepsia, and functional constipation—have not been completely understood through anatomical and biochemical models, and their complete treatment through allopathic medicines has not proven to be possible (Langmead and Rampton, 2001). Meanwhile, herbal medicines are widely used in many parts of the world to treat these disorders as an alternative (Langmead and Rampton, 2001). The liver is an integral organ of the GIT and herbal medicines are being researched to be used to treat many ailments of the liver, such as hepatitis and clotting problems. However, this research is still in the preliminary stages and several chemicals in these herbal medicines can be hazardous to health (Kim et al., 2020). The objective of this study is to make use the principles of ethnopharmacology to accentuate and understand the advantages of the herbal medicines in the treatment and prevention of GIT and liver diseases. Along with the uses, the toxicological effects of these medicines are also included in this study.

## Pre-clinical and clinical studies of herbal products in the management of GIT and liver diseases

GIT disorders contribute a major burden of diseases in the global healthcare system. A GIT disorder as common as diarrhea is one of the leading causes of mortality worldwide (Stickel and Schuppan, 2007). Although there are effective pharmacological drugs to treat most GIT ailments, a surging trend of resorting to more traditional forms of treatment (i.e., through herbal medication) has been noticed recently (Centers for Disease Control and Prevention, 2015).

Because significant pre-clinical and clinical research do not exist when it comes to the scientific effectiveness of herbal medicines, it is important to gather whatever scientific data that is available to substantiate the use of herbal medicines to treat different pathologies. The role of herbal medicines as the major treatment choice is extremely common in developing countries (Balick, 1996). According to research conducted by Tangjitman et al. (2015) in Thailand on the use of plant products for the treatment of various GIT ailments, various plants species and their different parts are used in Thailand to treat common GIT and liver problems, including diarrhea, flatulence, gastric ulcers, stomach ache, hemorrhoids, jaundice, and so on (Bodeker et al., 1997). Table 1 summarizes the use of different plant species for different GIT problems, along with their informant consensus factor (ICF) in this research (Bodeker et al., 1997; Tangjitman et al., 2015).

**TABLE 1:** The use of plant species for GIT problems, along with their informant consensus factor (ICF).

GIT disorders	Plant species used	ICF
Diarrhea	*Punica granatum* Linn (Punicaceae)	0.95
	*Psidium guajava* (Myrtaceae)	
	*Musa sapientum* (Musaceae)	
	*Leea indica* (Vitaceae)	
	*Ensete glaucum* (Musaceae)	
	*Celastrus paniculatus* (Celastraceae)	
	*Ochna integerrima* (Ochnacwae)	
Flatulence	*Zingiber montanum* (Zingiberaceae)	0.97
	*Zingiber ottensii* Valeton (Zingiberaceae)	
	*Boesenbergia rotunda* (L.) (Zingiberaceae)	
	*Kaempferia parviflora* (Zingiberaceae)	
Gastric ulcers	*Dillenia pentagyna* (Dilleniaceae)	0.92
	*Engelhardtia spicata* var. colebrookeana (Juglandaceae)	
	*Curcuma longa* (Zingiberaceae)	
	*Croton kongensis* (Euphorbiaceae)	
	*Ziziphus cambodiana* (Ziziphaceae)	
Mouth ulcers	*Melastoma malabathricum* (Melastomataceae)	0.95
Geographical tongue	*Melastoma malabathricum* (Melastomataceae)	1.00
Tooth ache	*Mussaenda sanderiana* (Rubiaceae)	0.50
Stomach ache	*Acorus calamus* (Acoraceae)	1.00
Constipation	*Ochna integerrima* (Ochnaceae)	1.00
Carminative	*Zingiber ottensii* (Zingiberaceae)	1.00
Food poisoning	*Ensete glaucum* (Musaceae)	1.00
Hemorrhoids	*Ziziphus cambodiana* (Ziziphaceae)	0.0
Laxative	*Senna* occidentalis (Fabaceae)	0.97
	*Euphorbia heterophylla* L. (Euphorbiaceae)	
	*Senna alata* (Fabaceae)	
	*Tamarindus indica* L (Fabaceae)	
Appetite enhancer	*Mussaenda sanderiana* (Rubiaceae)	1.00
Jaundice	*Gymnopetalum integrifolium* (Cucurbitaceae)	0.89
	*Dendrocalamus strictus* (Poaceae)	
	*Croton robustus* (Euphorbiaceae)	
	*Flemingia macrophylla* (Fabaceae)	
	*Mussaenda sanderiana* (Rubiaceae)	

It is generally believed that homeopathic or herbal medicines are without any considerable side effects and that they can be consumed without any fear of dangerous consequences on the body. However, this is far from the truth. Only a limited number of studies have been done on herbal products, which are also mostly inconclusive but list several side effects associated with these herbal medications. Table 2 lists a number of side effects of the plant species that are used in the treatment of GIT disorders (Tangjitman et al., 2015).

**TABLE 2:** A number of side effects of the plant species used for different GIT disorders (Tangjitman et al., 2015).

Plant species used for GIT disorders	Family	Toxicological effects
*Acorus calamus* L.	Acoraceae	Acute toxicity in mice
*Cassytha filiformis* L.	Lauraceae	Acute toxicity in mice
*Zingiber officinale Roscoe*	Zingiberaceae	Embryo toxic to pregnant rats
*Zingiber montanum (J.König) Link ex A.Dietr*	Zingiberaceae	Acute toxicity in mice
*Thunbergia laurifolia Lindl*	Acanthaceae	Decrease red blood cell in male mice
*Senna occidentalis* L.	Fabaceae	Intestinal disturbance in long-term used in rats
*Senna alata (L.) Roxb*	Fabaceae	Decrease hemoglobin and erythrocyte (RBC) count values in rats
*Euphorbia hirta* L.	Euphorbiaceae	Leukocytosis, dullness, anorexia, starry hair coat, and 20% mortality in rats
*Euphorbia heterophylla* L.	Euphorbiaceae	Increase leucopoenia in rats
*Kaempferia parviflora*	Zingiberaceae	Hepatotoxic to rats
*Flemingia macrophylla*	Fabaceae	Severe hypoglycemia followed by death within 24 h after administration to mice
*Celastrus paniculatus*	Celastraceae	Hyperactivity and loss of righting reflex in rats

Many herbal agents have been researched because of their potential use in the treatment of functional GIT disorders. For example, Kim et al. (2020) studied many herbal agents to identify their uses in the treatment of GIT diseases, such as STW-5 (Iberogast, liquid preparation of nine herbs), Mentha x Piperita (Lamiaceae), Rikkunshito (oral dried preparation of eight herbs), DA-9701 (Motilitone, a newer herbal drug from the seeds of *Pharbitis nil* Choisy (Convolvulaceae), and Corydalis tuber (Papaveraceae). These herbal medicines have been shown to treat GIT functional disorders such as irritable bowel disease, functional dyspepsia, gastroesophageal reflux disease, and constipation and used as analgesia and anti-spasmodics (Kim et al., 2020).

The liver receives most of its blood supply from the GIT’s circulation (Ebrahimi et al., 2020), so abnormalities affecting other areas of the GIT can have a huge implication on liver functionality and anatomy. Nonalcoholic fatty liver disease (NAFLD) is a highly prevalent condition, predominantly in people with obesity, diabetes, and hypertension. This disease has quite a dangerous sequela as it leads to nonalcoholic steatohepatitis (NASH). This eventually results in liver cirrhosis (the end-stage liver disease), which has no other treatment than liver transplant (Kim and Park, 2019; Xiao et al., 2019; Yan et al., 2020).

Various herbal medicines have been tested for use in the treatment of hepatic disorders, such as rhein, from *Rheum palmatum* L. (Polygonaceae), Huanglian Jiedu extract, Sho-saiko-to Juzen-taiho-to, and bofutsushosan, which are effective against NAFLD and NASH (Kim and Park, 2019; Xiao et al., 2019; Yan et al., 2020). Other herbal preparations have shown promising results in the treatment of liver ailments, such as like *Phyllanthus* L. (Phyllanthaceae), *Silybum marianum* (Asteraceae, milk thistle), *Glycyrrhiza glabra* (Fabaceae), and Liv 52 (mixture of herbs) (Dočkalová et al., 2018).

Coon and Ernst conducted a systematic review of the use of herbal medicines in chronic hepatitis C (Xiao et al., 2019). The authors cited 14 randomized clinical trials in view of the combined use of herbal products and interferon-alpha during antiviral treatment. Although there is difficulty in extrapolation and interpretation of results because of different methodological limits of the considered studies, the authors found that several herbal products and supplements (i.e., vitamin E, thymic extract, zinc, traditional Chinese medicine, *Glycyrrhiza glabra*, and oxymatrine) could exert potential virological and biochemical effects in the treatment of chronic hepatitis C infection because of a greater clearance of HCV-RNA and normalization of liver enzymes.

## Novel in vitro assays for the identification of potentially active compounds for the treatment of gastric and hepatic cancer

The cancers of GIT are very common throughout the world (Rasool et al., 2013; Abbas et al., 2017). Gastric and hepatic cancers are the leading causes of death across the world, gastric cancer being the fifth main cause of demise worldwide according to the World Health Organization (Rawla and Barsouk, 2019; Yang et al., 2022).

Gastric cancers are a group of complex cancers that require a multidisciplinary approach to be treated. The conventional mechanism to treat them requires surgical intervention, followed by radiological and chemotherapeutic treatment (Lelisho et al., 2022). Even after all these interventions, end stage cancers lead to death of patients in a very short time period. These interventions also have a toll on the psychological health of the patients, along with physical debility. Recently, attention toward research on herbal medications to treat gastric and other GIT cancers has significantly increased. Various phytochemicals (i.e., the active biochemical compounds present in plants used for health purposes) are being used and researched in GIT oncotherapy (Choudhari et al., 2020). Table 3 lists several phytochemicals, their anticancer characteristics, and their active compounds (Nakonieczna et al., 2020).

**TABLE 3:** A number of phytochemicals with their active compounds and anticancer characteristics (Nakonieczna et al., 2020).

Plant type	Family	Active metabolite	Anticancer characteristics
*Cordyceps cicadae*	Cordycipitaceae	Cordicepine	Enhances apoptosis
			Prevents metastasis
*Allium sativum* L.	Amaryllidaceae	Allicin	Enhances apoptotic
			Activity of cells
*Camellia sinensis*	Theaceae	Epigallocatechin gallate	Inhibits proliferation of cells
			Proapoptotic effect
			Reduces angiogenesis
			Anti-metastatic activity
*Cardiospermum halicacabum*	Sapindaceae	Synthesized gold nanoparticles	Apoptosis enhancing activity
*Plumbago zeylanica*	Plumbaginaceae	Plumbagin	Proapoptotic activity
			Anti-metastatic activity
			Autophagic activity
*Chrysosplenium nudicaule*	Saxifragaceae	TTF and DTFG	Apoptosis enhancing activity
			Reduces cell cycle activity
*Saussurea lappa*	Astreaceae	Costunolide	Inhibits proliferation of cells
			Proapoptotic effect
			Anti-metastatic action
*Nigella sativa* L.	Ranunculaceae	Thymoquinone	Proapoptotic effect
			Anti-metastatic action
			Causes sensitization of cells to chemotherapy
*Euphorbia lunulata*	Euphorbiaceae	Diterpenoids	Proapoptotic effect
			Anti-metastatic action
*Euphorbia esula*	Euphorbiaceae	Total extract	Proapoptotic effect
*Dioscorea bulbifera*	Dioscoreaceae	Diosbulbine B	Inhibits proliferation of cells
*Coptis chinesis*	Ranunculaceae	Berberine	Proapoptotic effect
			Anti-metastatic action
			Increased autophagic activity
*Stephania tetrandra*	Menispermaceae	Tetrandrine	Increased autophagic activity
			Proapoptotic effect
			Causes sensitization of cells to chemotherapy
*Piper longum*	Piperaceae	Piperlongumine	Inhibits proliferation of cells
			Proapoptotic effect
			Anti-metastatic action
*Sophora chrysophylla*	Fabaceae	Matrine	Inhibits proliferation of cells
			Proapoptotic effect
			Increased autophagic activity

Hepatocellular carcinoma has an exponentially increasing incidence with a high mortality rate (Fattovich et al., 2004). The trend of using herbal medications for the treatment of hepatic diseases is not a new concept. The use of herbal products to enhance the pharmacological and surgical treatment of liver cancer is widely studied and experimented upon. Herbal compounds such as *Curcuma longa* (Zingiberaceae), Resveratrol, *Silybum marianum* L. (Asteraceae), and Tanshinone have shown apoptotic and anti-proliferative effects, along with down-regulation of different compounds involved in metastatic cell growth and the arrest of the cell cycle at various stages (Lin et al., 2004).

## Beneficial and toxicological effects of herbal drugs in the light of ethnopharmacology

A number of the advantages of herbal medicines in the treatment of different pathologies of GIT and the liver have already been discussed (Carmona and Pereira, 2013). Some of the more general benefits of herbal medicines in enhancing the GIT system include how phytochemicals such as phytohemagglutinin (lectin) are involved in helping the gut to mature by stimulating intestinal growth (Mukonowenzou et al., 2021). The mechanism of action of this herbal drug was understood when administered to a rat’s gut, and it was shown to increase the number of crypts in the rat’s GIT (Mukonowenzou et al., 2021). Sangild et al. (2013) studied the importance of early-gut maturation and found that it enhances immunity, which protects children against lethal diseases such as necrotizing enterocolitis and post-weaning diarrhea.

Some of the toxicological effects of herbal medicines have been described earlier and it was shown that they can have hazardous effects. One of the common and note-worthy side effects of herbal medicines is their interaction with pharmacological drugs that are con-concurrently being consumed by the patients. Another important cause of the toxicological effects of herbal medicines is that the patients mostly use them as a self-medication tool. They tend to get them through unqualified practitioners and use them improperly without proper knowledge of their use (Fatima and Nayeem, 2016). A number of herbal medicines are reported to cause major hepatic dysfunctions such as acute hepatitis, hepatic failure, hepatorenal syndrome, and liver toxicity, particularly *Valeriana officinalis* (Valerianaceae), *Piper methysticum* G. (Piperaceae), *Cimicifuga racemose* (Ranunculaceae), *Scutellaria baicalensis* (Lamiaceae), *Larrea tridentate* (DC) (Zygophyllaceae), and *Stephaniae sinica* (Menispermaceae) (Licata et al., 2013; Quan et al., 2020).

## Conclusion

There is an ever-increasing trend toward the use of herbal medicines in both the developed and developing countries. Herbal medicines use natural products that are found in herbs and we know that many allopathic drugs contain these natural products in a refined manner. Although herbal medicines do exhibit some toxic effects, they can be very efficacious in treating a number of life-threatening conditions. However, unlike allopathic drugs, there is no authentic data available on their use, efficacy, toxicity, and side effects. Whenever a new pharmacological drug is introduced in the market, a large volume of information is made publicly available. Similarly, the herbal products that are in common use should be properly studied and experimented upon, and comprehensive data about their pharmacological aspects should be made publicly available.

There is this common myth that herbal medications have no to minimal side effects, so many people tend use them without any proper research and consultation. This can have very dangerous implications.

## Future implications

The fact that many herbal medicines have proven to be extremely beneficial against many life-threatening ailments means that it is very important to study them deeply in future. Special emphasis on the advancement of ethnopharmacology can prove to be a vital and excessively advantageous step toward the therapeutic realm of medicine. Future studies should be focused on placebo controlled, randomized, double-blind clinical trials, herbal product quality and standard criteria for diagnosis, treatment, outcome, and assessment of adverse herb reactions. This approach will provide insight into the risk and benefit profiles, which will hopefully be positive for at least some treatment modalities of herbal protagonists of modern herbal therapies. We can best face these promising challenges of pragmatic modern medicine by bridging the gap between the two medicinal cultures. This would be a tremendously prudent decision that will prove to enhance our existing healthcare system in the future.
